# A stochastic oscillator model simulates the entrainment of vertebrate cellular clocks by light

**DOI:** 10.1038/s41598-021-93913-2

**Published:** 2021-07-14

**Authors:** Vojtěch Kumpošt, Daniela Vallone, Srinivas Babu Gondi, Nicholas S. Foulkes, Ralf Mikut, Lennart Hilbert

**Affiliations:** 1 Institute for Automation and Applied Informatics, Karlsruhe Institute of Technology, Eggenstein-Leopoldshafen, Germany; 2 Institute of Biological and Chemical Systems-Biological Information Processing, Karlsruhe Institute of Technology, Eggenstein-Leopoldshafen, Germany; 3 Citrus Biotek, Hyderabad, India; 4 Centre for Organismal Studies Heidelberg, Ruprecht-Karls-Universität Heidelberg, Heidelberg, Germany; 5 Department of Systems Biology and Bioinformatics, Zoological Institute, Karlsruhe Institute of Technology, Karlsruhe, Germany

**Keywords:** Oscillators, Regulatory networks, Stochastic modelling, Systems biology, Cellular noise

## Abstract

The circadian clock is a cellular mechanism that synchronizes various biological processes with respect to the time of the day. While much progress has been made characterizing the molecular mechanisms underlying this clock, it is less clear how external light cues influence the dynamics of the core clock mechanism and thereby entrain it with the light–dark cycle. Zebrafish-derived cell cultures possess clocks that are directly light-entrainable, thus providing an attractive laboratory model for circadian entrainment. Here, we have developed a stochastic oscillator model of the zebrafish circadian clock, which accounts for the core clock negative feedback loop, light input, and the proliferation of single-cell oscillator noise into population-level luminescence recordings. The model accurately predicts the entrainment dynamics observed in bioluminescent clock reporter assays upon exposure to a wide range of lighting conditions. Furthermore, we have applied the model to obtain refitted parameter sets for cell cultures exposed to a variety of pharmacological treatments and predict changes in single-cell oscillator parameters. Our work paves the way for model-based, large-scale screens for genetic or pharmacologically-induced modifications to the entrainment of circadian clock function.

## Introduction

The circadian clock is a timekeeping mechanism that temporally coordinates the majority of biological processes according to the environmental day–night cycle^1^. At the molecular level, the core of this mechanism is based on a set of clock genes and proteins which constitute an autoregulatory, transcription–translation negative feedback loop^2^. In vertebrates, the positive elements Clock and Bmal are transcriptional activators that heterodimerize, bind to E-box enhancers, and thereby activate the transcription of a set of negative regulatory genes, which encode cryptochrome (Cry) and period (Per) proteins. Cry and Per directly interact and interfere with the Clock-Bmal activators, in consequence blocking transcriptional activation. This results in a reduction in *cry* and *per* gene expression, lowering of the nuclear levels of Per and Cry, leading ultimately to the release of inhibition of the Clock-Bmal heterodimers. This feedback cycle takes approximately 24 h to complete until it can start again, in the process generating an autonomous 24-h rhythm. Since the period of this rhythm is not precisely 24 h, it relies upon daily resetting to ensure synchronization with the environmental day–night cycle. Environmental signals that are indicative of the time of day (zeitgebers) are transduced to the core clock mechanism via various signaling pathways and thereby entrain the clock^3^. Light represents the primary zeitgeber in most organisms and accordingly has received significant attention^4^. In mammals, light is perceived by a dedicated circadian photoreceptor, the non-visual opsin melanopsin which is expressed in intrinsically photosensitive retinal ganglion cells (ipRGCs) in the retina^5^. Information on external light levels is then relayed indirectly via the retinohypothalamic tract (RHT) to the central clock located in the suprachiasmatic nucleus (SCN) within the hypothalamus, resulting in induced expression of the *per1* and *per2* clock genes, which in turn sets the phase of the SCN clock. The adjusted phase of the SCN clock is then communicated to other peripheral tissue clocks by a complex combination of systemic signals^6^. Frequent disruption of circadian clock function by exposure to irregular light–dark (LD) cycles can lead to mood and cognitive disorders^7^ as well as a broad range of human pathologies including metabolic disorders and cancer^8^.

Mathematical modeling has provided much insight into the regulatory mechanisms underlying the circadian clock, including the effects of pharmacological treatments and light entrainment^9^. Even though detailed mathematical models of the molecular mechanisms underlying circadian rhythms exist^10–13^, it is often favorable to use simpler models that condense the underlying mechanism into a few effective elements that are sufficient to reproduce experimentally observed behavior^14^. Such minimal models have previously been employed to study various aspects of the circadian clock, including light entrainment^15^, the effect of noise^16^, and the coupling of cellular feedback loops^17^. Intrinsic cellular noise leads to stochastic behavior of the circadian clock^18^. Even in bioluminescent reporter assays that record average signals from large cell populations, the noise in single-cell dynamics has a significant impact on clock rhythmicity when cells are placed in constant darkness (free-running conditions)^19^. In the absence of synchronizing zeitgebers, the single-cell oscillators progressively desynchronize, leading to the population-level loss of amplitude, even though individual cells maintain oscillation with undiminished amplitude^20^. Re-exposure to a zeitgeber has the effect of resynchronizing the asynchronous single-cell oscillators with the appropriate phase and rhythmicity re-emerges at the population level. Experimental measurement of the degree of single-cell clock synchronization requires long-term imaging of individual cells, which is technically challenging and not suited to large-scale screening experiments. Here, a mathematical modeling approach that could predict the degree of individual cell clock synchronization from population-level recordings would be valuable.

Fish represent attractive model organisms for studying circadian clocks in vertebrates. They constitute the largest and most diverse vertebrate group with species living under a wide range of environmental conditions. Therefore, they enable the study of how clocks have adapted to changing environmental conditions over the course of evolution^21^. Furthermore, a better understanding of circadian clock regulation and function in fish has potential benefits for aquaculture where lighting conditions have long been recognized to have a major impact on the rate of growth^22^. In addition, zebrafish is a particularly attractive genetic model organism to study the molecular basis of how the circadian clock is regulated by light. Specifically, zebrafish tissues and even cell lines possess directly light-entrainable clocks^23^. Unlike mammalian cells which require transient pharmacological treatments to synchronize cell culture clocks^24,25^, in the case of zebrafish cells, the clocks can be regulated non-invasively simply by changing lighting conditions. These light-responsive cell lines are also suitable for high-throughput screening as well as studies of the transcriptional control mechanisms mediating light entrainment^26^. In this regard, many bioluminescent reporter systems have now been established in zebrafish cell lines and enable the non-invasive assessment of dynamic changes in clock gene transcription at high temporal resolution over the course of light exposure protocols^27^. A previous theoretical model of the zebrafish circadian clock has shown that, in principle, simulated single-cell oscillations can be used to reconstruct average luminescence signals from cell populations^28^. However, this model is based on the consideration of two interlocked feedback loops and a large number of parameters, making reliable fitting and readjustment of model parameters to limited experimental data difficult.

Here, we present a minimal stochastic oscillator model of the circadian clock in zebrafish cell lines. This model, based on the Kim–Forger model^13^, contains only three variables but reproduces and predicts the main oscillatory characteristics observed in bioluminescent reporter assays under a broad range of experimental lighting conditions. We further adjusted the model parameters to infer the impact of various pharmacological treatments on core clock dynamics at the cellular level. Our work thus provides a tool to characterize core clock dynamics, with the ability to provide hypotheses on how cell population-level luminescence signals under various lighting and pharmacological protocols might emerge from single-cell behavior. Subsequently, based on these hypotheses, more refined, single-cell imaging experimental approaches can be used on a much smaller scale to validate the screening results. This paves the way towards model-based, large-scale screens for genetically or pharmacologically-induced modifications affecting the degree of synchronization of single-cell circadian oscillators.

## Results

### Characterization of light entrainment in cell culture

**Figure 1:**
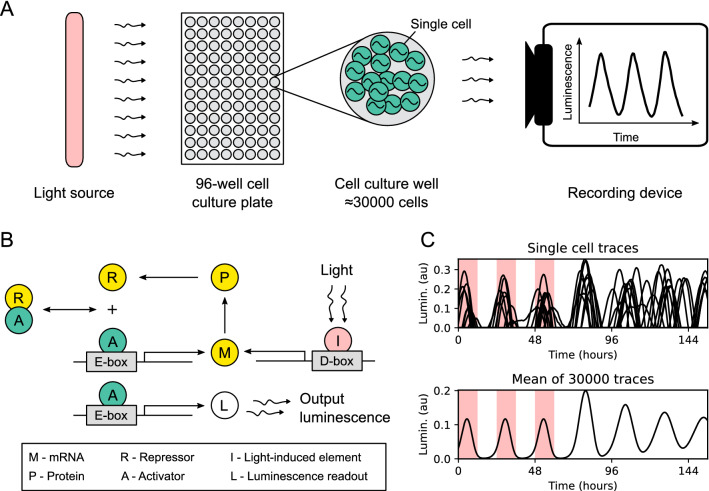
Experiment setup and simulation of core circadian clock dynamics in zebrafish cell cultures. (**A**) 96-well culture plates were placed in a dark room and illuminated with a time-controlled light source. Each well contains approximately 30,000 cells transfected with a bioluminescent reporter of *zper1b* transcription. The output luminescence from each well is recorded as a separate time trace. (**B**) Schematic of the mathematical model of the zebrafish core circadian clock. The activator (green) binds to the E-box enhancer in the promoter of a clock gene and activates the production of the repressor (yellow). After transcription, translation, and translocation back to the nucleus, the repressor binds to the activator, thus preventing E-box-driven transcriptional activation. External light stimuli take effect through the activation of a light-driven gene with the D-box enhancer. The luminescence output is assumed to be proportional to the E-box activation. (**C**) Simulated luminescence traces are obtained by averaging 30,000 independent evaluations of the model. The output luminescence after normalization is presented in arbitrary units (au). Red shading indicates periods when the light source was turned on.

To explore the entrainment dynamics of the vertebrate circadian clock in an experimental model, we used zebrafish PAC-2 cell lines. PAC-2 cellular clocks can be entrained by direct exposure to light and they have been widely used to study the transcriptional mechanisms of light entrainment^26,29,30^. We chose to study the dynamics of circadian clock gene expression by non-invasive luminescence assays of PAC-2 cells stably transfected with a *zper1b* promoter bioluminescent reporter construct^26^. In these cells, reporter gene transcription is directly controlled by the core clock mechanism via E-box enhancers. To obtain a data set that supports the development, adjustment, and validation of a mathematical model, we recorded luminescence over several days, in cells exposed to a variety of different lighting conditions (Fig. 1A and Supplementary Fig. S1), including various LD cycles, constant darkness, and constant light. The resulting data set contains an extensive number of repeats (Supplementary Table S1), further supporting model fitting and validation.

### Stochastic oscillator model

As a conceptual basis for the development of a mathematical model for the PAC-2 cell clock we used a negative feedback function that represents regulation by protein sequestration (Kim–Forger model)^13^. This model considers that the transcription-translation negative feedback loop at the core of the zebrafish circadian clock consists of positive elements (activators) and negative elements (repressors). The activators bind to E-box enhancers located in the promoter region of clock genes, thus inducing the transcription of the repressors. After transcription, translation, and translocation back to the nucleus, the repressors physically interact with the activators and thereby inhibit the transcriptional induction of their own genes^31^. As a first step, we describe the core clock mathematically using three coupled differential equations connected in a negative feedback loop [Fig. 1B, Eq. (3)]. The three state variables implicitly encode the delay that is necessary to produce the oscillatory behavior^32^ and thereby provide a minimal model of the circadian clock^33,34^. To more closely connect our mathematical model to the known regulatory processes, we arbitrarily label the three variables as mRNA (*M*), cytoplasmic protein (*P*), and nuclear protein that acts as a repressor (*R*). In the Kim–Forger model, the repressor *R* binds to the activator *A*, forming an inactive *RA* complex with 1:1 stoichiometry, thus preventing the binding of the activator to the E-box enhancers, resulting in inhibition of gene expression. We used an according protein sequestration function [${\text {f}}$, Eq. (2)] to describe the transcriptional activation^13,35^. This function defines the transcriptional activation as a function of the repressor and activator concentrations. The light input is implemented as a light-induced element *I*, whose activation increases the production of mRNA *M*. Similar extensions to the minimal models of the circadian clock are commonly used to represent light stimuli^15,36,37^. In our model, the additive light term *I* represents light-driven activation of an additional set of light-driven *per* and *cry* clock genes that is characteristic of the zebrafish circadian clock and is mediated by D-box enhancers^29,30^. These light-driven genes are specifically activated by light with minimal activation in darkness, and thus most accurately represented by an additive term. As a result of this gene activation, there is an increase in the production of negative elements. The function ${\text {f}}$ is the quantity in our model that most closely represents the induction of the *zper1b:luc* reporter by E-box enhancers. We thus use the value of the function ${\text {f}}$ to represent the luminescence read-out obtained in our experiments.

Each well of a 96-well culture plate contains approximately 30,000 independently oscillating cells that contribute to the luminescence signal measured from that well. Intrinsic cellular noise affects the individual oscillators but is averaged in the population-level luminescence recordings^20^. One classical phenomenon demonstrating the relevance of single-cell dynamics is the progressive loss of the amplitude of oscillations at the population level in constant darkness while individual cells continue to oscillate with undiminished amplitude (Fig. 1C). We have therefore implemented noise at the single-cell level by additive noise terms in our model^16^. To mimic the averaging over a population of single-cell oscillators in the luminescence assays, we simulate 30,000 instances of our model and obtain the mean value as the final output^19,28^.

### Parameter adjustment and model validation

**Figure 2:**
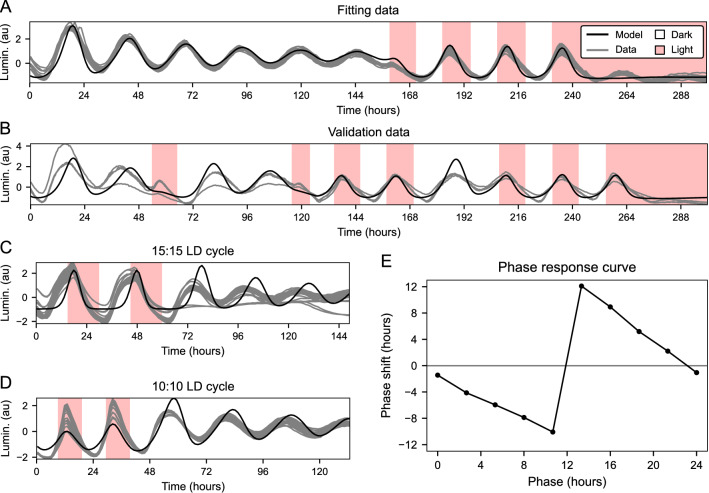
Simulation of *zper1b:luc* expression dynamics under various lighting conditions. (**A**) On this plate, cell cultures were exposed to 6 days of constant darkness, three 12:12 LD cycles, and 3 days of constant light. This recording was used to estimate the model parameters. (**B**) On this plate, cell cultures were exposed to a combination of LD cycles, constant darkness, and constant light. The same parameters as in (**A**) were used to run the model, showing that the model can also reproduce dynamics of data not used for parameter fitting. (**C**) This plate was exposed to 15:15 LD cycles. Parameters estimated from (**A**) were used without further fitting. (**D**) This plate was exposed to 10:10 LD cycles. Parameters estimated from (**A**) were used without further fitting. (**E**) Simulated phase response curve to 12-h light pulses.

We adjusted the model parameters to luminescence recordings with a custom fitting algorithm based on an evolutionary optimizer. Specifically, we compared the behavior of our model with luminescence recordings showing three characteristic phenomena: attenuation of the oscillation amplitude during free-running conditions in constant darkness, synchronization of the phase of the rhythm upon transfer from constant darkness to an LD cycle, and oscillation repression under constant light. We first adjusted our model parameters to luminescence recordings with varied light conditions and evaluated the goodness of fit by the model efficiency coefficient [$E_f$, Eq. (15)]. The resulting model efficiency was high (16 wells, mean standard deviation, $E_f = 0.89 \pm 0.03$, Fig. 2A). Without further readjustment of model parameters, we simulated luminescence traces for experiments with different lighting conditions, resulting in fair model efficiency (4 wells, $E_f = 0.63 \pm 0.21$, Fig. 2B). Upon visual inspection, the model captured all key behaviors observed in *zper1b:luc* assays. We also tested the performance of the model under LD cycles with period lengths significantly longer and shorter than 24 h (30 h, 15:15 LD; and 20 h, 10:10 LD). In the case of our model, the goodness of fit was lower than under the 12:12 LD cycle (32 wells, 15:15 LD, $E_f = 0.24 \pm 0.20$, Fig. 2C; 16 wells, 10:10 LD, $E_f = 0.60 \pm 0.11$, Fig. 2D), mainly due to mismatched profiles of each expression cycle. Nevertheless, the model was still entrained and oscillated with the corresponding period of these long and short LD cycles. This is consistent with our previous experiments where we showed that the PAC-2 cell clock exhibits rhythmicity under these conditions with an adjustment of the period length of the reporter rhythm to match the period of the LD cycle^26^. We also simulated the phase response curve (PRC) for the fitted model and 12-h long light pulses (Fig. 2E). The resulting type 0 PRC^38^ is also consistent with our previous experiments showing that the PAC-2 cell culture exhibits a type 0 PRC^26^. Therefore, the model can reproduce and predict characteristics of the entrainment of the endogenous PAC-2 cellular clock under complex lighting conditions and loses its accuracy only for entraining period lengths that differ significantly from the natural 12:12 LD cycle.

We also investigated the effect of varying noise intensity ($\sigma$) on the population-level luminescence signal, using the model with fitted parameters (Supplementary Fig. S2). The most obvious effect of increasing $\sigma$ is a more pronounced desynchronization of the individual oscillators, resulting in a faster loss of amplitude at the population level in constant darkness. However, a noticeable change can be also observed under the LD cycle. With increasing noise, individual peaks have a smaller amplitude and a wider profile.

### Representing effects of pharmacological treatments as model parameter changes

**Figure 3:**
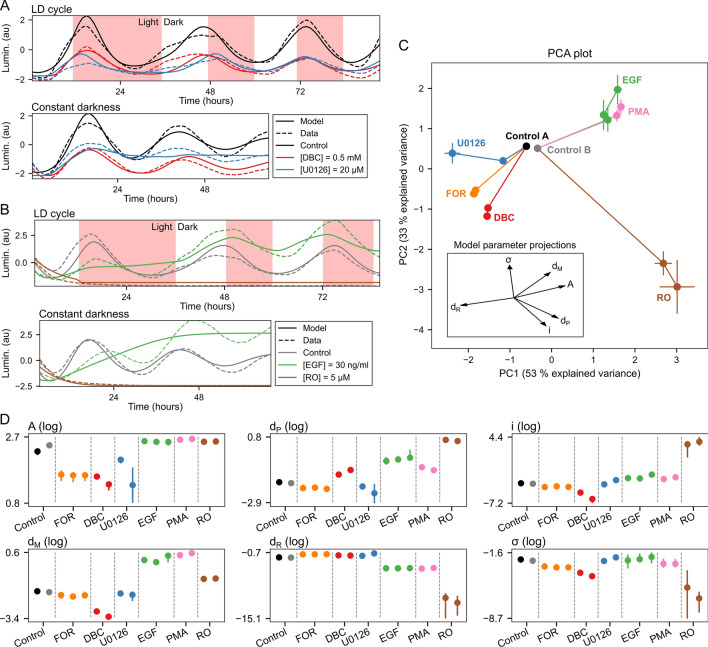
Characterization of compound effects by refitting of model parameters. (**A**) Representative experimental data traces and model fits for plate set A. Plate set A contained treatments with dimethyl sulfoxide (DMSO) control, forskolin (FOR), dibutyryl cAMP (DBC), and U0126. See Supplementary Fig. S4A–C for model fits for all concentrations of FOR, DBC, and U0126. (**B**) Representative experimental data traces and model fits for plate set B. Plate set B contained treatments with DMSO control, epidermal growth factor (EGF), phorbol-12-myristate-13-acetate (PMA), and ro-318220 (RO). See Supplementary Fig. S4D–F for model fits for all concentrations of EGF, PMA, and RO. (**C**) The principal component plot depicts the projection of the compound-dependent changes in model parameters on two main principal components (PCs), which provides a tool to visualize the changes of the model parameters in a two-dimensional plot. Each compound and concentration is represented with a point. The error bars at each point indicate the spread of the final population of parameter sets obtained by a differential evolution algorithm (median ± median absolute deviation). Individual values used to calculate the median points are shown in Supplementary Fig. S3. Lines connect increasing concentrations of the same compound, starting from the control condition. Control A is a DMSO control for FOR, DBC, and U0126. Control B is a DMSO control for PMA, EGF, and RO. The explained variance for the first three PCs was 53%, 33%, and 8%. (**D**) Parameter values for all pharmacological treatments. Each dot indicates median value and lines maximal and minimal values of the parameter sets obtained by a differential evolution algorithm. Multiple dots per compound indicate increasing concentrations of the same compound from left to right.

We next wished to explore whether our mathematical model could discriminate between changes in basic properties of the clock mechanism induced by different pharmacological treatments. We treated our clock reporter PAC-2 cells with six compounds that affect three major signaling pathways regulating the core clock. Specifically, we used forskolin (FOR) and dibutyryl cAMP (DBC) as activators of the cAMP pathway; epidermal growth factor (EGF) and U0126 as an activator and an inhibitor of the MAPK pathway, respectively, as well as phorbol-12-myristate-13-acetate (PMA) and ro-318220 (RO) as an activator and an inhibitor of the PKC pathway, respectively. All these pathways have been previously implicated in light entrainment^39–42^. We assessed the effect of the exposure to these compounds under two different sets of lighting conditions (Fig. 3A,B). Starting with the model parameters from our initial adjustment to data obtained from untreated cells, we executed a step-wise refitting to the results after pharmacological treatments. Each compound was administrated at three different concentrations (Supplementary Table S1), and parameters for the individual compound concentrations were estimated iteratively starting with the lowest compound concentrations and finishing with the highest compound concentrations. We set the threshold for a good fit as $E_f > 0$, which excluded 4 of 20 treatments from our further analysis. The accepted and excluded fits are listed in Supplementary Table S2. The final model fits for all pharmacological treatments are depicted in Supplementary Fig. S4.

The principal component analysis (PCA) of the parameter sets representing the individual compounds revealed that the two main principal components together account for 86% of the explained variance. We can therefore depict the relative changes of the six free parameters in the two-dimensional space defined by the two main principal components (Fig. 3C). In the PCA plot, every compound exhibits a change away from the control conditions. Compound-specific changes were increasingly pronounced at higher concentrations, while the overall direction of parameter change was conserved for each of the compounds. Based on the direction of displacement in the principal component plot, different compounds can be grouped. FOR, DBC, and U0126 are all associated with a decrease in principal component 1, reflected in a loss of the oscillation amplitude that is similar for all three compounds (Fig. 3A, Supplementary Fig. S4A–C). PMA and EGF are associated with an increase in principal component 1, reflected in a decreased amplitude of the oscillations during the first day of the recording, followed by a sudden transition to a higher amplitude in the subsequent days of the assay (Fig. 3B, Supplementary Fig. S4D,E). RO showed a distinct decrease in principal component 2 and lies considerably further away from all other compounds. This reflects the complete absence of oscillatory behavior associated with this specific treatment (Fig. 3B, Supplementary Fig. S4F). Taken together, our refitting approach allows the categorization of these compounds based on their inferred effects on the model parameters, as indicated by the proximity of compounds with similar effects in the PCA plot.

Beyond the overview provided by the PCA plot, we could also relate changes in individual parameter values to changes in the luminescence traces upon pharmacological treatments (Fig. 3D). For example, all compounds leading to a decrease in oscillatory amplitude (FOR, DBC, U0126) exhibit a decrease in parameter *A*, representing activator concentration. In contrast, compounds resulting in higher amplitude (EGF, PMA) exhibit an increase in *A*. In the case of RO, a compound that abolishes oscillatory behavior, the parameter $d_R$ is decreased. $d_R$ represents the degradation rate of the repressor, so the decrease of $d_R$ indicates that a stabilization of the repressor drives constant inhibition of the transcription activation. Leaving aside the fully repressed case of RO, the parameter *i* (light sensitivity) seems positively correlated with the parameter $\sigma$ (noise intensity). This relationship suggests that entrainment due to higher light sensitivity can counteract the faster desynchronization of individual oscillators that results from higher noise intensity. This balance between light sensitivity and noise strength allows the possibility that FOR, DBC, and U0236, even though they all similarly decrease the amplitude of oscillation, have different noise intensities. Note that our analysis implies that the compounds typically result in a change of several model parameters, making our model unable to identify the exact regulatory element within the clock mechanism targeted by a given compound.

### Treatment-specific mechanisms of amplitude loss

**Figure 4:**
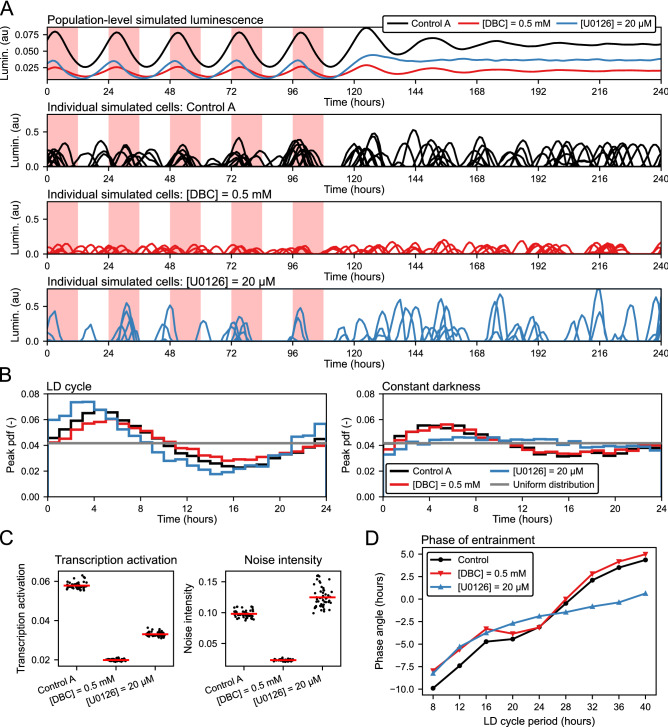
The population-level loss of amplitude is caused by different underlying single-cell mechanisms. (**A**) Output of the model under 5 days of LD cycle followed by 5 days of constant darkness. For each compound the population-level mean is displayed with 10 of the 30,000 individual traces from which the mean was calculated. (**B**) Histograms showing probability density function (pdf) of phases (peak positions of individual traces) under LD cycle and constant darkness (times between 72 and 96, and 122 and 146 hours of panel **A**). (**C**) Transcription activation was calculated as mean of the output luminescence in constant darkness. The values for control ($L = 0.0576 \pm 0.0017$) are higher than the values for DBC ($L = 0.0198 \pm 0.0004$) and U0126 ($L = 0.0331 \pm 0.0012$). The noise intensity value for control ($\sigma = 0.098 \pm 0.006$) was lower than for U0126 ($\sigma = 0.124 \pm 0.016$) but higher than for DBC ($\sigma = 0.023 \pm 0.001$). Mean and standard deviation were calculated across the final population of parameter sets obtained by the evolutionary optimizer. (**D**) Simulated effect of the LD cycle period (T) on the phase of entrainment. The phase angle is calculated as the time interval between the light onset and the oscillation peak normalized by T/24. Negative values indicate that the oscillation peak is delayed with respect to the light onset.

The impact of the FOR, DBC and U0126 compounds on the rhythmic parameters of the luminescence recordings is similar. However, close inspection of the PCA plot reveals that U0126 lies further away from FOR and DBC (Fig. 3C). This relative displacement appears to be mainly in the direction of parameter $\sigma$, suggesting that U0126 differs from FOR and DBC by higher noise intensity, as is also supported by our assessment of changes in individual parameters (Fig. 3D). As our model consists of an ensemble of oscillators, we wondered if the observed difference can be mapped to the altered behavior of the simulated individual cells. As an illustrative example, we focused on comparing the treatments with 0.5 mM DBC and 20 $\upmu$M U0126. When synchronized cell populations were transferred to constant darkness, both treatments resulted in a more rapid loss of population amplitude than in control-treated cultures. At the level of single cells, however, our model suggests that DBC treatment resulted in reduced single-cell oscillator amplitude, while incubation with U0126 caused a more pronounced desynchronization of individual oscillators due to higher noise (Fig. 4). We have investigated this effect of differences in the noise intensity further by visualizing the histograms of phases of individual cells (Fig. 4B). During the LD cycle, histograms of phases in all three cases (Control, DBC, U0126) markedly differ from the uniform distribution, indicating synchronized oscillations. After transfer into the constant darkness, the histogram of phases for U0126 is more uniformly distributed than the one of control and DBC treatment, indicating a more pronounced loss of synchrony. Taken together, our simulations imply two distinct sets of single-cell dynamics that both result in the accelerated loss of rhythm amplitude at the population level.

To further assess how the capacity for entrainment by light differs upon DBC and U0126 treatment, we extracted the phase of entrainment from simulations paced by LD cycles with different periods (T). In this analysis, we applied various T and calculated the phase angle as the time interval between the light onset and the oscillation peak^38^ (Fig. 4D). We could not construct conventional PRCs because, for some treatments, oscillations in constant darkness diminished too rapidly, making an analysis based on luminescence peaks impossible. Our analysis revealed that, for T smaller than 24 h both compounds, DBC, and U0126, reduce the phase angle relative to the control treatment. For T higher than 24 h, DBC had no apparent effect, while U0126 markedly reduced the phase angle. Considering that these simulated phase angle curves provide highly specific predictions for future experiments, we generated the same type of curves for the other compounds (Supplementary Fig. S5). Interestingly, these simulations predict that the entrainment dynamics upon U0126 treatment are similar to those upon EGF and PMA treatment, even though the loss of amplitude upon U0126 treatment under a 24-h LD cycle is closer to the results obtained upon DBC and FOR treatment.

## Discussion

Here, we present a stochastic oscillator model that mimics the dynamic properties of the core circadian clock in cultures of zebrafish cell lines. We find that the model accurately recapitulates the rhythmic properties and entrainment of luminescence recordings under a range of lighting conditions, only losing accuracy when simulating the effects of exposure to LD cycles with periods significantly longer or shorter than 24 h. Furthermore, we demonstrate the utility of our model for the characterization of luminescence recordings in terms of changes in single-cell core clock regulation.

Our stochastic model is based on the three-variable Kim–Forger model^13^ extended by light input and noise terms. This model differs from the more common Goodwin model^33^ by using a protein sequestration function, instead of the Hill function, to model transcription inhibition. The sequestration function explicitly represents interactions of the repressor and activator proteins, and exhibits quantitatively different dynamics from the Goodwin model^35^. It was also suggested that transcriptional repression in multicellular organisms is accomplished via protein sequestration rather than cooperative binding reactions described by the Hill function^43^. Computationally, the protein sequestration function is more reliable for stochastic simulations^44^. The good fit to our data suggests that the protein sequestration function is indeed a plausible representation of transcription inhibition also in zebrafish and works reliably for the stochastic simulations in our study.

Minimal models of the core circadian clock were used in many previous studies to investigate the properties of circadian entrainment^36,45–47^. Our work shows that such a minimal model can simulate the key characteristics of the zebrafish circadian clock. The model simplifies the precise molecular details of clock regulation and so loses predictive strength in experiments where cells are exposed to light cycles with periods that deviate significantly from the natural 12:12 LD cycle. Another inaccuracy our model exhibits is an inability to reproduce oscillation in constant darkness for some of the compounds (U0126, EGF, PMA), even though the model can very precisely capture the dynamics under an LD cycle. This is mainly the case for higher concentrations of U0126, for which the oscillation amplitude is low, as well as for EGF and PMA, which show strong transient behavior after compound administration. One simplification in our model that is potentially relevant in this respect is the use of only a single feedback loop, even though the actual circadian clock consists of multiple interlocked feedback loops^31^. Furthermore, the core feedback loop consists of multiple clock genes with overlapping functions, which has been predicted to contribute to the regulatory robustness of the clock^48^. However, our results indicate that at a systems level, these extra clock genes effectively act within the context of a complex regulatory circuit that behaves as a single feedback loop.

Cellular noise is an intrinsic part of the clock mechanism^18^ and it is essential to consider this factor when interpreting observations made at the cell population level. In our model, the presence of noise allows us to reproduce the desynchronization of phases in constant darkness. This can be seen, for example, for the treatment with compounds DBC and U0126: single cells show sustained oscillations in constant darkness, while differences in synchronization lead to differences in the population-level luminescence signal (Fig. 4A). In the case of untreated cells, the necessity to address single-cell dynamics is illustrated by experiments that show sustained oscillations in single cells of a population without oscillations at the population level^20^. In our experimental setup, cells presumably function as independent oscillators and are implemented accordingly in our model. However, in vivo in various zebrafish tissues, cells can be coupled with each other and such an inter-cellular coupling may be predicted to affect entrainment properties^36,37^.

The importance of cellular noise was also recognized by a previous model of the zebrafish circadian clock^28^. As in our model, signals from an ensemble of stochastic oscillators were averaged to simulate population-level luminescence recordings. We reduced the number of variables (from 5 to 3) and the number of free parameters (from 15 to 6) in relation to the previous model, enabling the application of a fitting algorithm to adjust and quantitatively validate our model against experimental data. The possibility to fit our model to luminescence recordings further provides the foundation for inference of changes in core clock parameters resulting from pharmacological treatments or genetic changes. We used the refitting of our model to understand the effect of different compounds on the core clock dynamics. Considering that treatments result in combined changes in several parameters (Fig. 3D), treatment effects appear to be multi-causal and difficult to reduce to a single parameter change. Also, our approach of using a minimal model does not identify the specific molecular interactions that are affected by a given treatment, but rather provides a tool to reveal effective changes in the dynamic properties of the core clock mechanism as an overall regulatory system. This approach is conceptually consistent with previous work suggesting that all light-transduction signaling pathways converge to the same active element^49^ and thus exhibit similar effects on the overall clock dynamics.

Inspecting the resulting parameter space, we found that the parameter sets representing the treatments with FOR and DBC were closer to each other than other compounds. This is expected, as both FOR and DBC act as activators of the cAMP pathway and should thus similarly affect the core clock mechanism^50^. The parameter sets representing EGF and PMA were also close to each other. This is consistent with previous work showing that the PKC pathway (activated by PMA) is an upstream activator of the MAPK pathway (activated by EGF)^39,51^. Those results indicate that our model as well as the fitting procedure can also accurately classify changes in core clock parameters. In addition, our model can predict changes in single-cell dynamics from the population-level luminescence recordings, as seen for the DBC and U0126 treatments. The close proximity of the parameter values for DBC and U0126 lead to similar effects at the population level, namely a reduced amplitude of rhythmic reporter gene expression. However, our model predicts that the effect of U0126 is substantially different from the effect of DBC at the level of the single-cell simulations. While our model predicts that DBC causes the loss of population-level amplitude by inhibition of gene expression at the single-cell level, U0126 is predicted to cause a more rapid desynchronization of the single-cell oscillators.

We employed PCA to map multiple parameter sets into a single plot. The PCA plot contains no additional information with respect to the luminescence time courses themselves, but allows us to easily assess an arbitrary number of experimental conditions in a single image. In a screen with a small number of compounds and concentrations, it is still possible to assess luminescence time courses by eye to compare the effects of individual compounds. Our method, however, would still allow this kind of assessment even when much larger numbers of compounds were tested, for example, as part of a high throughput screen. Here, an assessment of experimental traces by eye would be possible in principle, but difficult in practice, and subjective biases would be prevented by the application of our model. The PCA plot also provides a rapid visual validation for our refitting approach, as it shows that parameter values that represent similar treatment effects are in fact placed close to each other.

The effect of pharmacological treatments strongly depends on environmental light conditions^52–54^. We, therefore, simulated how treatment with different compounds affects the entrainment by LD cycles of different periods (Fig. 4D, Supplementary Fig. S5). We found that the phase angle under different LD cycles is similar for control and cAMP inhibitors FOR and DBC. While U0126 in our experiments showed similar effects on population-level luminescence as DBC and FOR, the predicted phase angle to different LD cycles is closer to EGF and PMA. Interestingly, U0126 is an inhibitor of the MAPK pathway, the same pathway that is positively regulated by EGF and PMA.

Assessment of the degree of synchronization of single-cell oscillators in a cell population in culture involves technically challenging and time-consuming imaging that is not trivial to perform as part of a large-scale screen. Within this context, our modeling approach provides the possibility to make rapid predictions about the behavior of individual cell clocks from population-level luminescence recordings. These predictions could then be followed up by more refined imaging-based assays of single-cell dynamics on a much smaller set of samples. Therefore, our mathematical model should contribute a rapid and scalable tool for interpreting the effects of pharmacological treatments and genetic modifications on the circadian clock at the cellular level.

## Methods

### Luminescence recordings

We used PAC-2 light-responsive cells stably transfected with the *zper1b:luc* reporter^26^. 96-well culture plates were seeded with approximately 30,000 cells per well and placed in a dark room. Different lighting conditions were applied by exposure to a time-controlled white light source (Supplementary Fig. S1). Approximately every 40 min, each plate was automatically moved into the counting chamber of a TopCount NXT counter (Perkin Elmer) and luminescence was measured for approximately 5 min (3 s per well). Forskolin (FOR), dibutyryl cAMP (DBC), epidermal growth factor (EGF), U0126, phorbol-12-myristate-13-acetate (PMA), and ro-318220 (RO) were used at concentrations indicated in Supplementary Table S1. An overview of the experiment design is shown in Fig. 1A.

### Mathematical model

Our model is based on the Kim–Forger model^13^ that represents oscillatory behavior of three clock elements denoted here as mRNA *M*, protein *P*, and repressor *R*. The full model reads

1a $$\begin{aligned} \frac{\mathrm {d}M}{\mathrm {d}t}&= v_M {\text {f}}(R, A, K) - d_M M \end{aligned}$$

1b $$\begin{aligned} \frac{\mathrm {d}P}{\mathrm {d}t}&= v_P M - d_P P\end{aligned}$$

1c $$\begin{aligned} \frac{\mathrm {d}R}{\mathrm {d}t}&= v_R P - d_R R\end{aligned}$$

1d $$\begin{aligned} {\text {f}}(R, A, K)&= \frac{A - R - K + \sqrt{\left( A - R - K \right) ^2 + 4AK}}{2A}, \end{aligned}$$

where *A* denotes activator concentration, *K* is a dissociation constant of the *RA* complex, $v_*$ are production rates, and $d_*$ are degradation rates. In our fitting procedure, both the experimental data and model output are normalized. We thus nondimensionalize amplitudes of the model following variable scaling introduced by Kim and Forger^13^ which leads to rescaling of production rates $v_*$ to 1. This reduces the number of parameters by 3 effectively avoiding over-parametrization and connected problems of non-unique results of the fitting.

To further reduce the number of parameters, we note that the model gives oscillatory behavior only if *K* is sufficiently low ($10^{-4}$ or smaller). In the limit $K \rightarrow 0$ the function ${\text {f}}$ can be approximated by a piece-wise function^13,35^

2 $$\begin{aligned} {\text {f}}(R, A) = {\left\{ \begin{array}{ll} 1 - \frac{R}{A} &{} \frac{R}{A} \le 1 \\ 0 &{} \frac{R}{A} > 1 \\ \end{array}\right. }. \end{aligned}$$

Supplementary Figure S9 shows that the model output does not effectively change by assuming $K \rightarrow 0$ for small $K \le 10^{-4}$.

Finally, we extend the nondimensionalized model by light input and noise terms to obtain the model used in our study. The final set of equations reads

3a $$\begin{aligned} \frac{\mathrm {d}M}{\mathrm {d}t}&= {\text {f}}(R, A) - d_M M + i I + \sigma \xi _M\end{aligned}$$

3b $$\begin{aligned} \frac{\mathrm {d}P}{\mathrm {d}t}&= M - d_P P + \sigma \xi _{P}\end{aligned}$$

3c $$\begin{aligned} \frac{\mathrm {d}R}{\mathrm {d}t}&= P - d_R R + \sigma \xi _{R}, \end{aligned}$$

where *I* is a light input function, which is 0 in darkness and 1 in light. Parameter *i* represents the light sensitivity of the clock. $\sigma$ is noise intensity, and $\xi _*$ are independent Wiener processes.

The luminescence produced by a single cell is then equal to

4 $$\begin{aligned} L_j = {\text {f}}(R, A). \end{aligned}$$

The final model output that corresponds to the luminescence output of a cell culture well is

5 $$\begin{aligned} L = \frac{1}{n}\sum _{j = 1}^{n} L_j, \end{aligned}$$

where *j* are independent evaluations of the stochastic model and $n = 30{,}000$.

### Data and model output normalization

Normalization of luminescence recordings is necessary to eliminate variations in amplitude caused by inherent experimental error. We normalized data from untreated cell lines on a trace-by-trace basis as Z-scores defined as

6 $$\begin{aligned} {\text {z}}(x) = \frac{x - {\bar{x}}}{S_x}, \end{aligned}$$

where ${\bar{x}}$ and $S_x$ are sample mean and standard deviation of trace *x*. As the normalized traces of repeats showed very low variance, we used their mean for model fitting and validation (Supplementary Fig. S6).

For the plates with compound-treated cell lines, we could not use the trace-by-trace Z-score normalization as the change in mean and variance can be an important aspect of the compound effect. Therefore, we defined the adjusted Z-score

7 $$\begin{aligned} {{\mathrm{z}}_{\mathrm{d}}}(x) = \frac{x - {\bar{x}}}{S_x} \frac{S_d}{S_c} + \frac{{\bar{x}}_d - {\bar{x}}_c}{S_c}, \end{aligned}$$

where ${\bar{x}}$ and $S_x$ are sample mean and standard deviation of trace *x*, ${\bar{x}}_d$ and $S_d$ are mean of means and mean of standard deviations for all traces of the corresponding compound, and ${\bar{x}}_c$ and $S_c$ are mean of means and mean of standard deviations for all control-treated traces. For control the equation reduces to the standard Z-score because $S_d = S_c$ and ${\bar{x}}_d = {\bar{x}}_c$, which means that the values for control can be directly compared to the values of untreated cell lines from other plates normalized by a standard Z-score. Visual comparison of raw and normalized data showed low variance in traces of the same compound while correctly preserving the relative changes of the compound traces to control (Supplementary Figs. S7 and S8).

Model output was also normalized to correspond to the normalized data. For untreated cell lines, we used Z-scores as defined in Eq. (6). For compound-treated cell lines, we used the adjusted Z-score whose form for the model output is

8 $$\begin{aligned} {\text {z}}_{\mathrm{{model}}}({\hat{x}}) = \frac{{\hat{x}} - {\bar{x}}_0}{S_0}. \end{aligned}$$

Here, ${\hat{x}}$ is model output, and ${\hat{x}}_0$ and $S_0$ are sample mean and standard deviation of the simulation output for the control parameter set. Equation (8) can be derived directly from Eq. (7). The model output is averaged over multiple evaluations, which leads to low variance between repeated population-level simulations. Therefore, we set ${\bar{x}}_d = {\bar{x}}$, $S_d = S_x$, ${\bar{x}}_c = {\bar{x}}_0$, and $S_c = S_0$ which directly reduces Eqs. (7) to (8).

### Optimization algorithm

The proposed mathematical model contains 6 free parameters: activator concentration (*A*), degradation rates ($d_M$, $d_P$, $d_R$), light sensitivity (*i*) and noise intensity ($\sigma$). We adjusted those parameters to the untreated and compound-treated luminescence recordings in four consecutive steps using a differential evolution optimizer^55^. First, the noise was set to 0 and other parameters were estimated based on general properties of the circadian clock known from the literature. Second, we estimated noise based on the damping ratio observed in the luminescence recordings. Third, the model parameters were fine-tuned to the luminescence recordings of untreated cell lines. Fourth, we further adjusted the model parameters to the compound-treated cell lines. We developed this step-wise optimization approach to minimize the number of computationally expensive evaluations of the stochastic model, thus enabling the fitting of the stochastic model to a wide variety of experimental data. To further reduce the computational effort during optimization, we constructed the mean trace from 1000 trajectories instead of 30,000. Supplementary Figure S10 shows that this simplification has minimal effect on the final mean. In the following, we describe the different steps of this fitting approach in detail.

#### Fitting the model in the absence of noise

In the first step of the fitting algorithm, a custom cost function was used to find a population of parameter sets that can reproduce basic properties of a circadian oscillator known from literature^56^. Those properties are oscillations with an approximately 24-h period in constant darkness and entrainment by external LD cycles^20^. As the probability of the oscillations is the highest when the degradation terms are equal^57^, we introduced a constrain on equal degradation terms

9 $$\begin{aligned} d = d_m = d_p = d_r. \end{aligned}$$

The cost function for optimization reads

10 $$\begin{aligned} C_1 = \left( T_{\mathrm{DD}} - 24 \right) ^2 + \left( T_{\mathrm{LD}} - 24 \right) ^2 + \left( P_{\mathrm {LD}} - P_{\mathrm {LDs}} + 12 \right) ^2, \end{aligned}$$

where $T_{\mathrm{DD}}$ is period in constant darkness (DD), $T_{\mathrm {LD}}$ is period under 12:12 LD cycle, $P_{\mathrm{LD}}$ is phase of the oscillator under LD, and $P_{\mathrm {LDs}}$ is phase of the oscillator under LD with 12-h phase shift. The first two terms of the equation ensure circadian oscillations in constant darkness and under 12:12 LD cycle respectively. The third term ensures that the model reacts to different phases of the LD cycles^56^. The optimization was repeated 50 times to obtain 50 different parameter sets. Those are subsequently used as an initial population for the next fitting step.

#### Estimating noise intensity

Higher noise intensity causes faster desynchronization of individual oscillators, which leads to faster damping of oscillation amplitude in the population-level recordings^19^. We estimated the damping ratio from a damped sine fit to data. The function for the damped sine is

11 $$\begin{aligned} {\text {f}}_{\mathrm {ds}}(t) = A \mathrm {e}^{-d t} \sin \left( \frac{2 \pi t}{T} + \theta \right) \end{aligned}$$

with amplitude *A*, damping ratio *d*, period *T*, and phase $\theta$.

We estimated the corresponding value of the noise intensity parameter $\sigma$ for each of the 50 parameter sets obtained in the previous fitting step. In this step, only the noise intensity was estimated while all other parameters of the model were fixed to the values estimated previously. The cost function for the differential evolution optimization is

12 $$\begin{aligned} C_2 = \left( d_\text {data} - d_\text {model} \right) ^2, \end{aligned}$$

where $d_\text {data}$ is the damping ratio estimated from the luminescence recordings (first six days of the fitting data, Fig. 2A) and $d_\text {model}$ is the damping ratio estimated from the model output (simulated in constant darkness after prior entrainment by 12:12 LD cycle).

#### Parameter adjustment for the untreated cell lines

To numerically find the optimal parameter set for a specific luminescence recording, we used differential evolution with the initial population given by the previous step of the optimization algorithm. As a cost function, we used the squared error

13 $$\begin{aligned} C_3 = \frac{1}{n}\sum _{i = 1}^{n} \left( x_i - {\hat{x}}_i \right) ^2, \end{aligned}$$

where $x_i$ denotes data points and ${\hat{x}}_i$ denotes the output of the model. The initial conditions were estimated by letting the model run under 12:12 LD cycles for 10 days. In this way, we diminished the effect of arbitrary initial conditions on the final solution. Further, we assumed that untreated cells produce sustained circadian oscillations in constant darkness^20^. Therefore, we checked the solution of the deterministic model ($\sigma = 0$) for the presence of sustained oscillations in constant darkness by detecting the amplitudes and locations of peaks. If no oscillations were found for the corresponding parameter set, the cost function returns its maximal value without the need to additionally evaluate the stochastic model with noise terms. Based on the assumption of sustained oscillations, we also kept the previously imposed constraint on the equality of degradation terms.

#### Parameter adjustment for the compound-treated cell lines

The cost function for experiments with pharmacological treatments is

14 $$\begin{aligned} C_4 = \frac{1}{n}\sum _{i = 1}^{n} \left( x_i - {\hat{x}}_i \right) ^2 + \frac{1}{m}\sum _{i = 1}^{m} \left( y_i - {\hat{y}}_i \right) ^2, \end{aligned}$$

where $x_i, y_i$ denote luminescence recordings recorded under the LD cycle and in constant darkness respectively and ${\hat{x}}_i$, ${\hat{y}}_i$ denote model outputs for the LD cycle and constant darkness respectively. For compound-treated cell lines, we could not assume that the cells exhibit oscillations in constant darkness. Therefore, we relaxed the constraint on equal degradation rates and did not test the deterministic model for the presence of sustained oscillations.

The compounds were administered right before the beginning of the recording which caused transient behavior observable in the data. To incorporate this into our simulations, we precalculated initial conditions by running the model of the untreated cells for 10 days and then taking the last values as initial conditions for the simulations of pharmacological treatments. The resulting initial conditions correspond to the state of the oscillator at the transition from light to dark. This corresponds to the experimental data used in our work that start at the beginning of the dark phase. The initial conditions were fixed for the whole process of optimization. Individual compound doses were fitted iteratively from control to the highest concentration of the specific compound. At each step, the final optimized population from the previous step was used as the initial population for the next step.

### Goodness of fit metric

We assessed the goodness of fit using the model efficiency coefficient defined as^58^

15 $$\begin{aligned} E_f = 1 - \frac{\sum _{i=1}^{n}\left( x_i - {\hat{x}}_i \right) ^2}{\sum _{i=1}^{n}\left( x_i - {\bar{x}} \right) ^2}, \end{aligned}$$

where $x_i$ are data points, ${\hat{x}}_i$ are corresponding model outputs and ${\bar{x}}$ is data mean. The values of the model efficiency coefficient can range from $-\infty$ to 1. Values near 1 indicate high predictive value of the model while negative values indicate that data mean ${\bar{x}}$ is a better predictor than model output ${\hat{x}}$.

### Software implementation

All code was written in Julia^59^ version 1.6. For the numerical solution of the differential equations, we used the DifferentialEquations.jl package^60^. The fitting algorithm was implemented using differential evolution from the BlackBoxOptim.jl package. All data and code needed to replicate the results and generate the paper figures are available at https://github.com/vkumpost/biolum.

## Supplementary Information

Supplementary Information.
