# Supplementary material for: A stochastic oscillator model simulates the entrainment of vertebrate cellular clocks by light
Source: Sci Rep. 2021 Jul 14;11:14497. doi: 10.1038/s41598-021-93913-2 (PMC8280200; doi:10.1038/s41598-021-93913-2)
Supplement: Supplementary file 1 — Supplementary Information. [file 41598_2021_93913_MOESM1_ESM.pdf]

A stochastic oscillator model simulates the entrainment of vertebrate cellular clocks by light – supplementary information

Vojtěch Kumpošt[1,2], Daniela Vallone[2], Srinivas Babu Gondi[3], Nicholas S. Foulkes[2,4,*], Ralf Mikut[1,*], and Lennart Hilbert[2,5,*]

[1]Institute for Automation and Applied Informatics, Karlsruhe Institute of Technology, Eggenstein-Leopoldshafen, Germany
[2]Institute of Biological and Chemical Systems - Biological Information Processing, Karlsruhe Institute of Technology, Eggenstein-Leopoldshafen, Germany
[3]Citrus Biotek, Hyderabad, India
[4]Centre for Organismal Studies Heidelberg, Ruprecht-Karls-Universität Heidelberg, Heidelberg, Germany
[5]Zoological Institute, Department of Systems Biology and Bioinformatics, Karlsruhe Institute of Technology, Karlsruhe, Germany
[*]nicholas.foulkes@kit.edu; ralf.mikut@kit.edu; lennart.hilbert@kit.edu

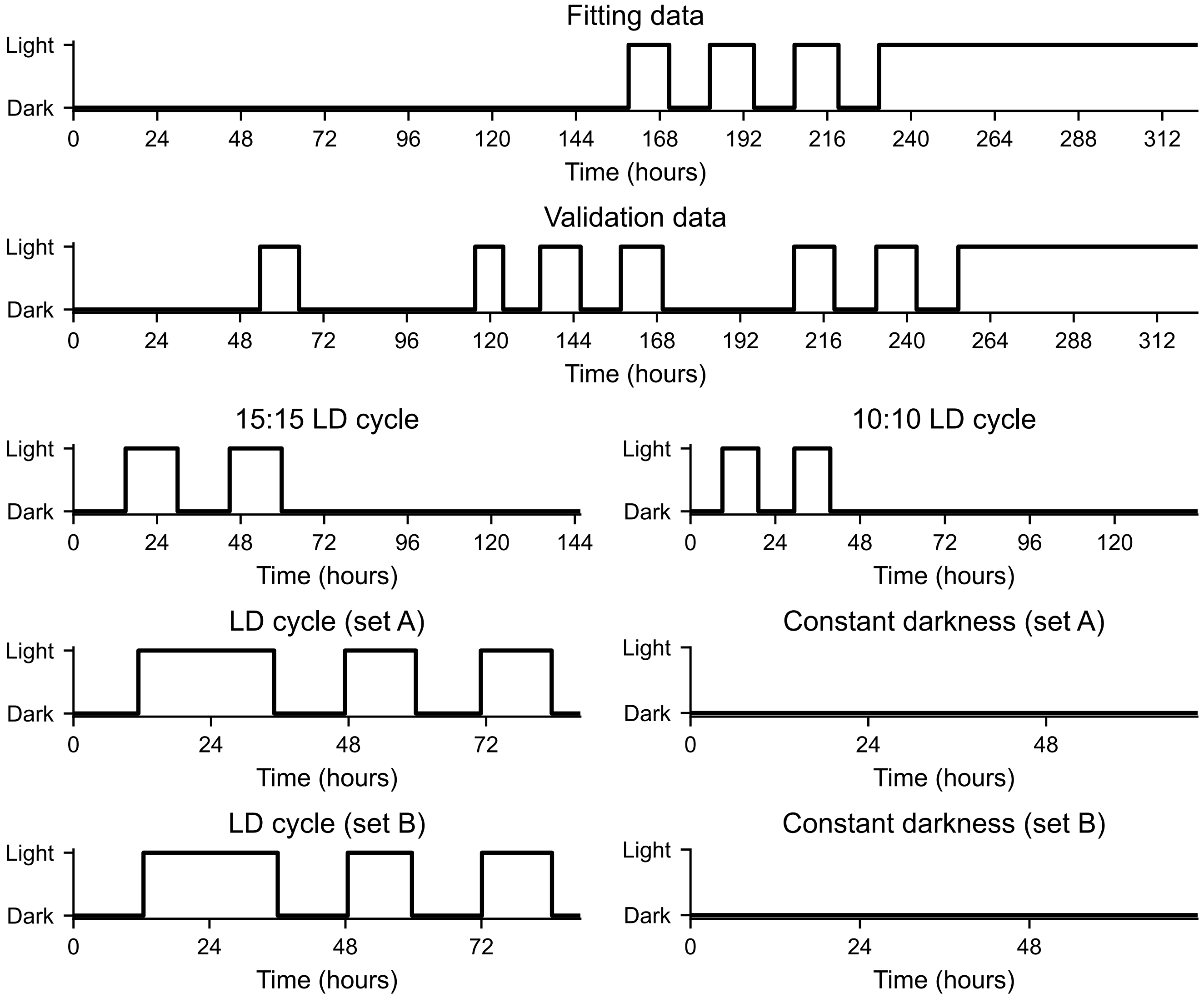

Supplementary Figure S1. Various lighting conditions were used to study the light entrainment of the zebrafish cell cultures. Fitting data was obtained by exposing untreated cell lines to constant darkness for six days, followed by three days of the 12:12 LD cycle, and finally placed under constant light for three days. Validation data were recorded for 14 days under various lighting conditions, including LD cycles and periods of constant light and darkness. 15:15 LD cycle and 10:10 LD cycle data were recorded under two 15:15 and 10:10 LD cycles, respectively, followed by a period of constant darkness. Tested compounds were split into two plate sets (A and B). Each plate set consisted of two plates, one was placed under a light regime with a 24-hour phase reversal light pulse followed by two days of 12:12 LD cycle and the other was placed for three days in constant darkness.

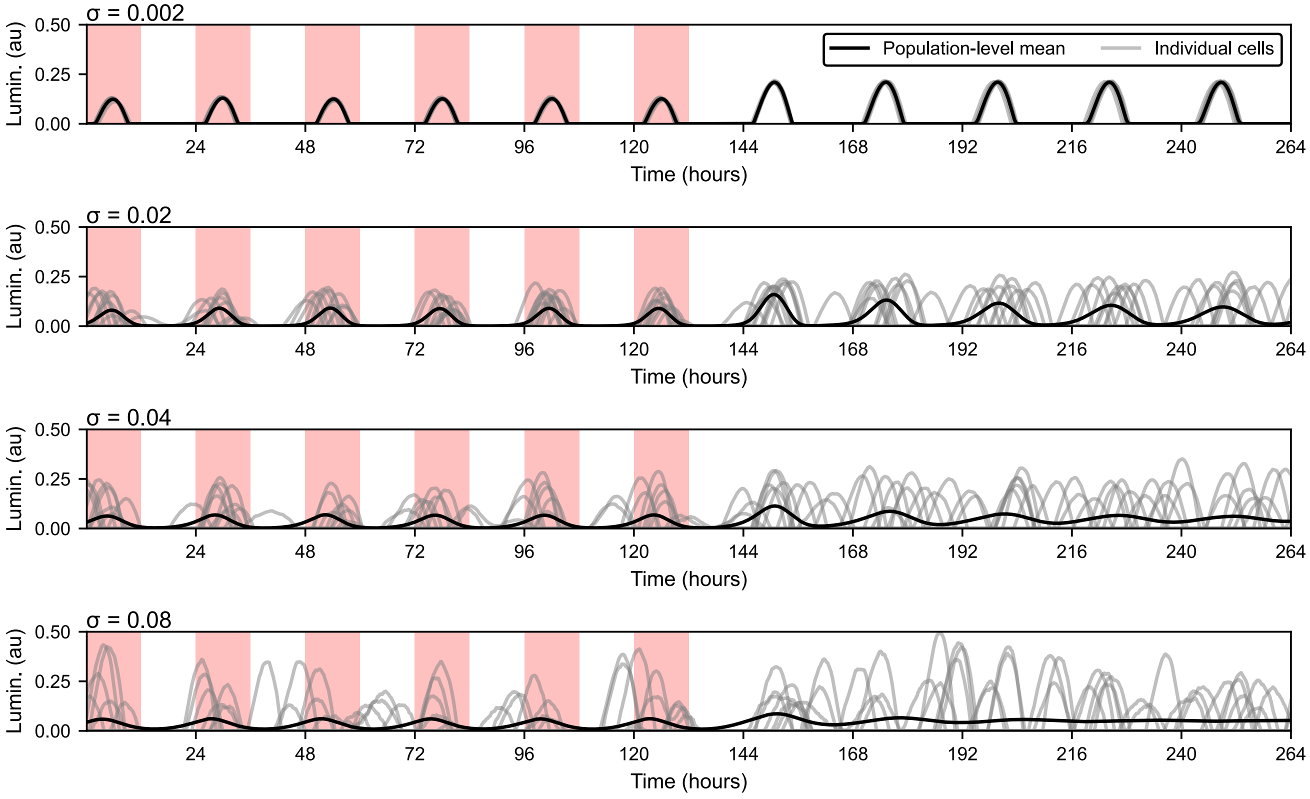

Supplementary Figure S2. Effect of noise intensity on light entrainment and desynchronization in constant darkness. We compared the model output for noise intensities 0.002, 0.02, 0.04 (fitted value to untreated cells), and 0.08. Increasing noise intensity leads to a more pronounced desynchronization of the individual oscillators, resulting in a faster loss of amplitude at the population level in constant darkness. A noticeable change can be also observed under the LD cycle. With increasing noise, individual peaks have a smaller amplitude and a wider profile.

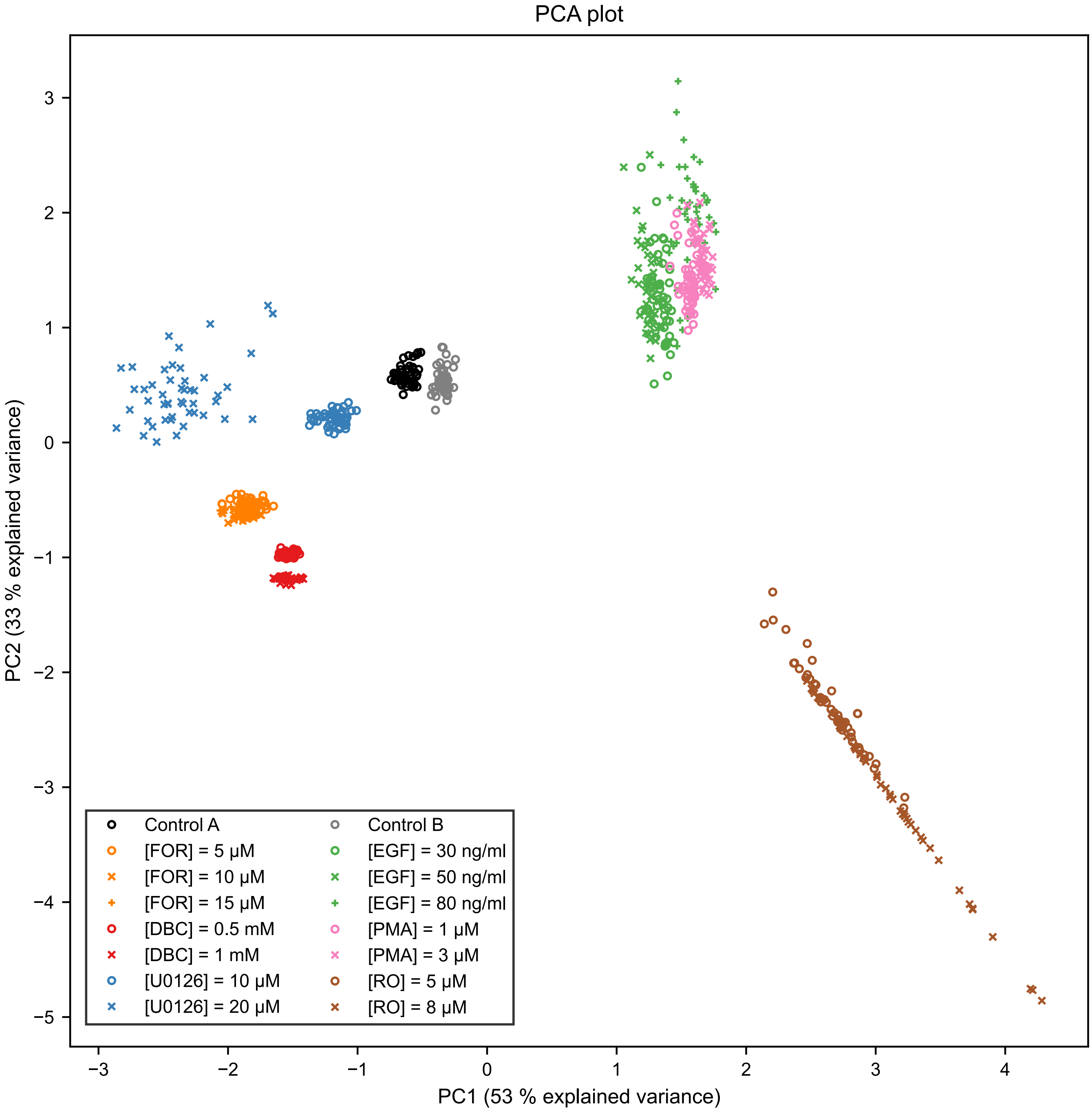

Supplementary Figure S3. Individual values in the PCA analysis. This plot is equivalent to Figure 3C showing individual points for each compound concentration. Each point represents one member of the final population obtained from running a differential evolution algorithm to find the best parameter fit for the data. The population size was 50.

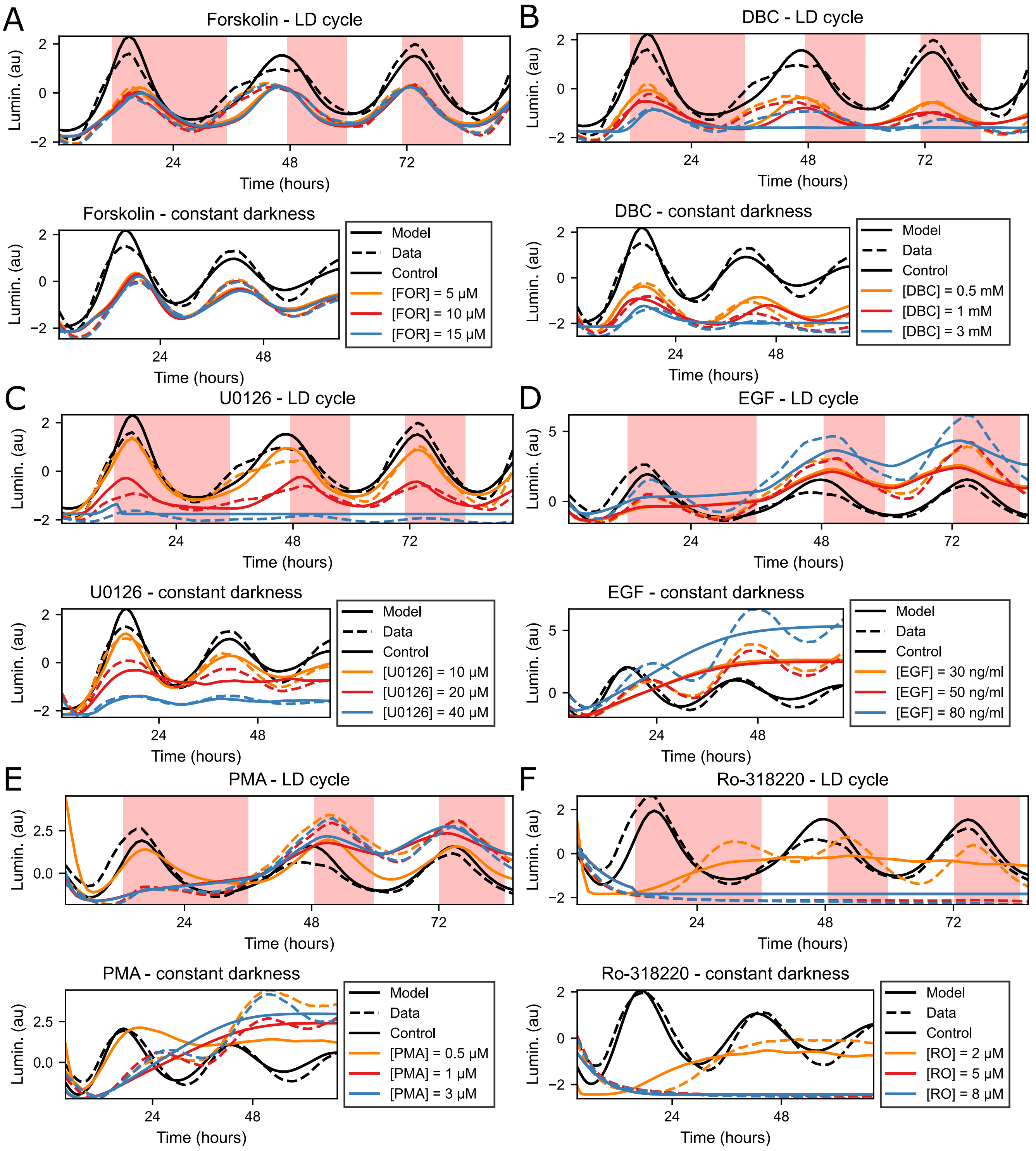

Supplementary Figure S4. Model fit to pharmacological treatments. The model parameters were fitted to 6 compounds, each of which was applied in 3 different concentrations. The corresponding goodness of fit estimations can be seen in Supplementary Table S2. (A) Model fit to treatments with forskolin (FOR). (B) Model fit to treatments with dibutyryl cAMP (DBC). (C) Model fit to treatments with U0126. (D) Model fit to treatments with epidermal growth factor (EGF). (E) Model fit to treatments with phorbol-12-myristate-13-acetate (PMA). (F) Model fit to treatments with ro-318220 (RO).

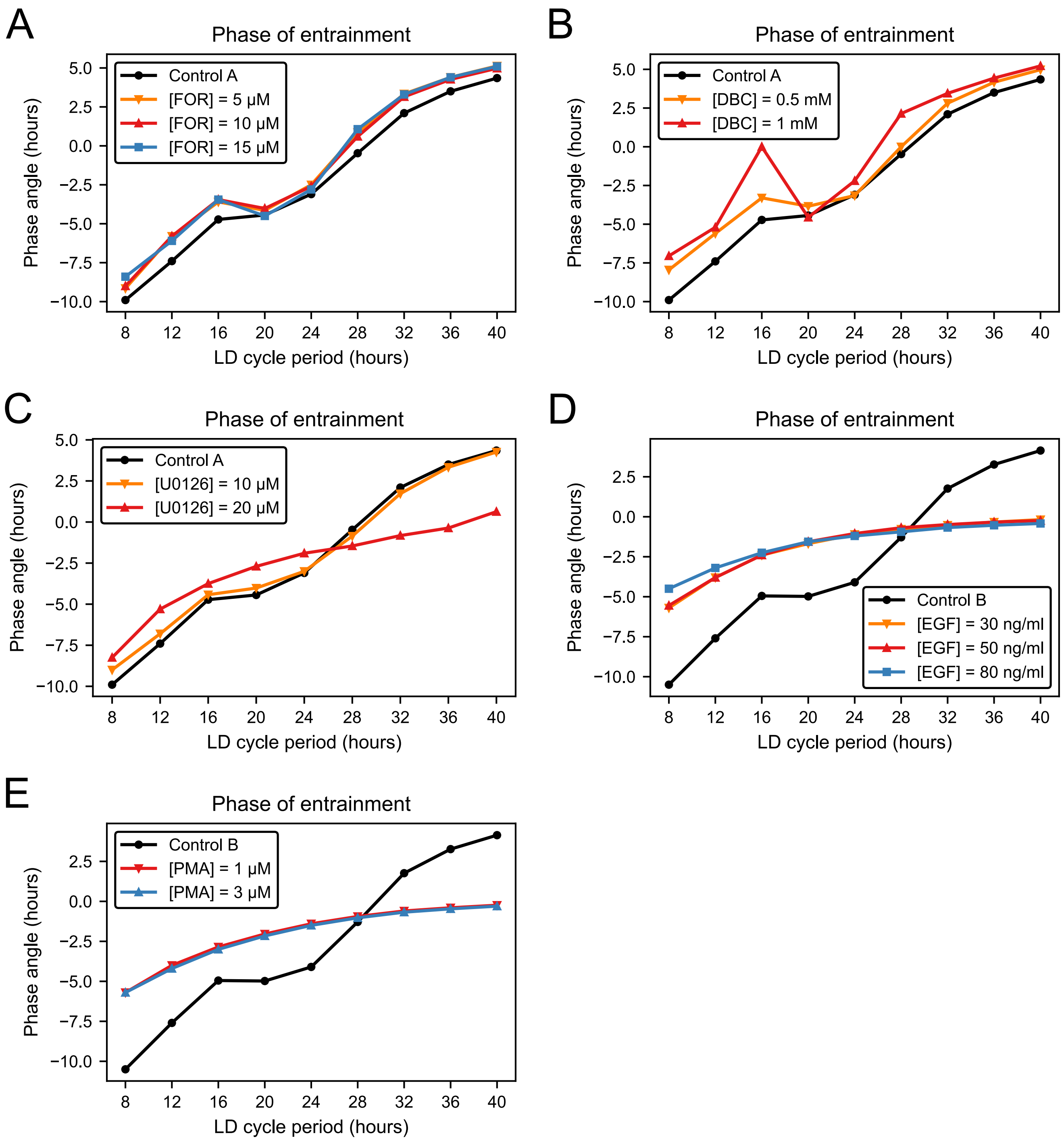

Supplementary Figure S5. The phase of entrainment depends on the LD cycle period (T) and administrated compound. The phase angle is calculated as the time interval between the light onset and the oscillation peak normalized by T/24. Negative values indicate that the oscillation peak is delayed with respect to the light onset. (A) forskolin (FOR) (B) dibutyryl cAMP (DBC) (C) U0126 (D) epidermal growth factor (EGF) (E) phorbol-12-myristate-13-acetate (PMA).

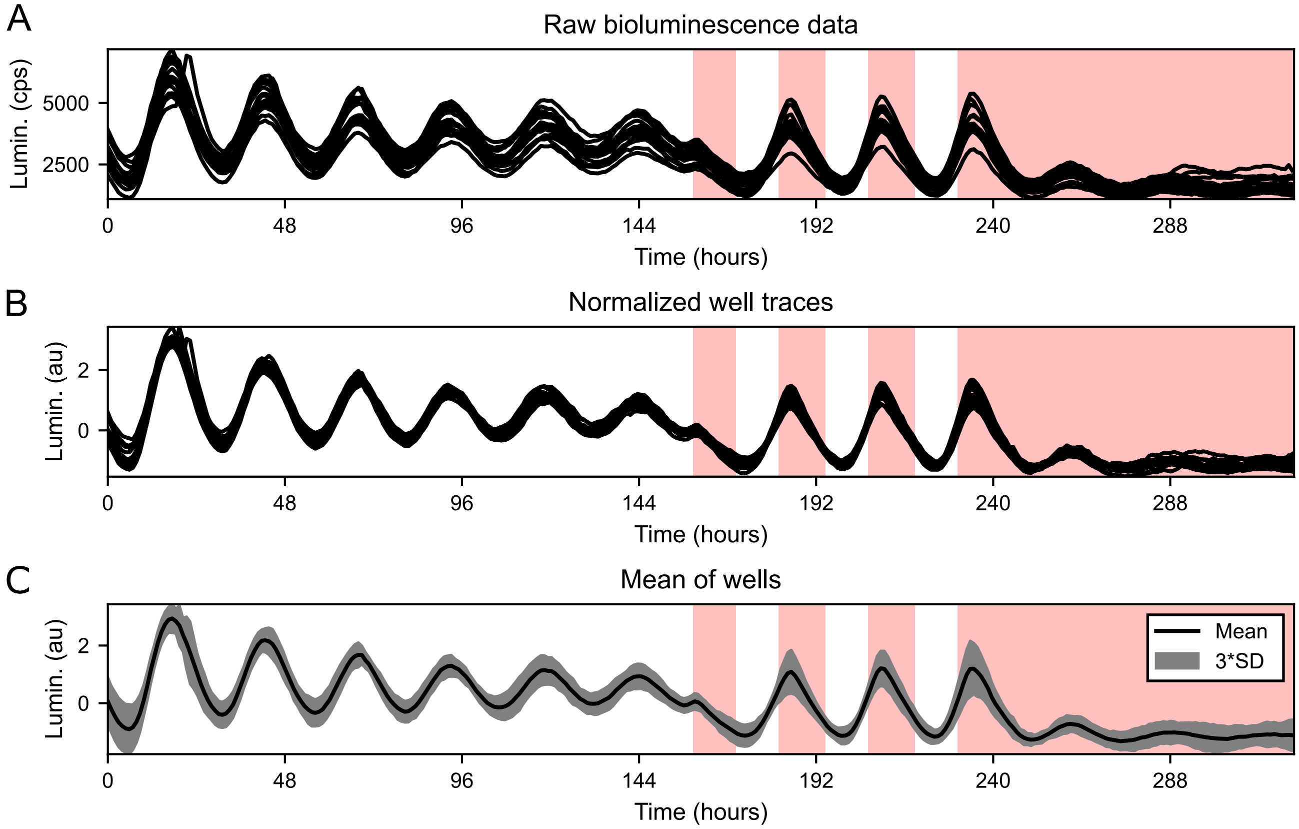

Supplementary Figure S6. Normalization decreases variance in luminescence recordings. (A) Raw luminescence recordings in counts per second (cps) were obtained from 16 cell culture wells. (B) After Z-score normalization, the variance among the individual traces is reduced and the resulting traces are presented in arbitrary units (au). (C) The individual normalized traces were used to calculate a mean value that we consequently used to fit and validate the model. The gray area around the mean shows 3 * standard deviation (SD).

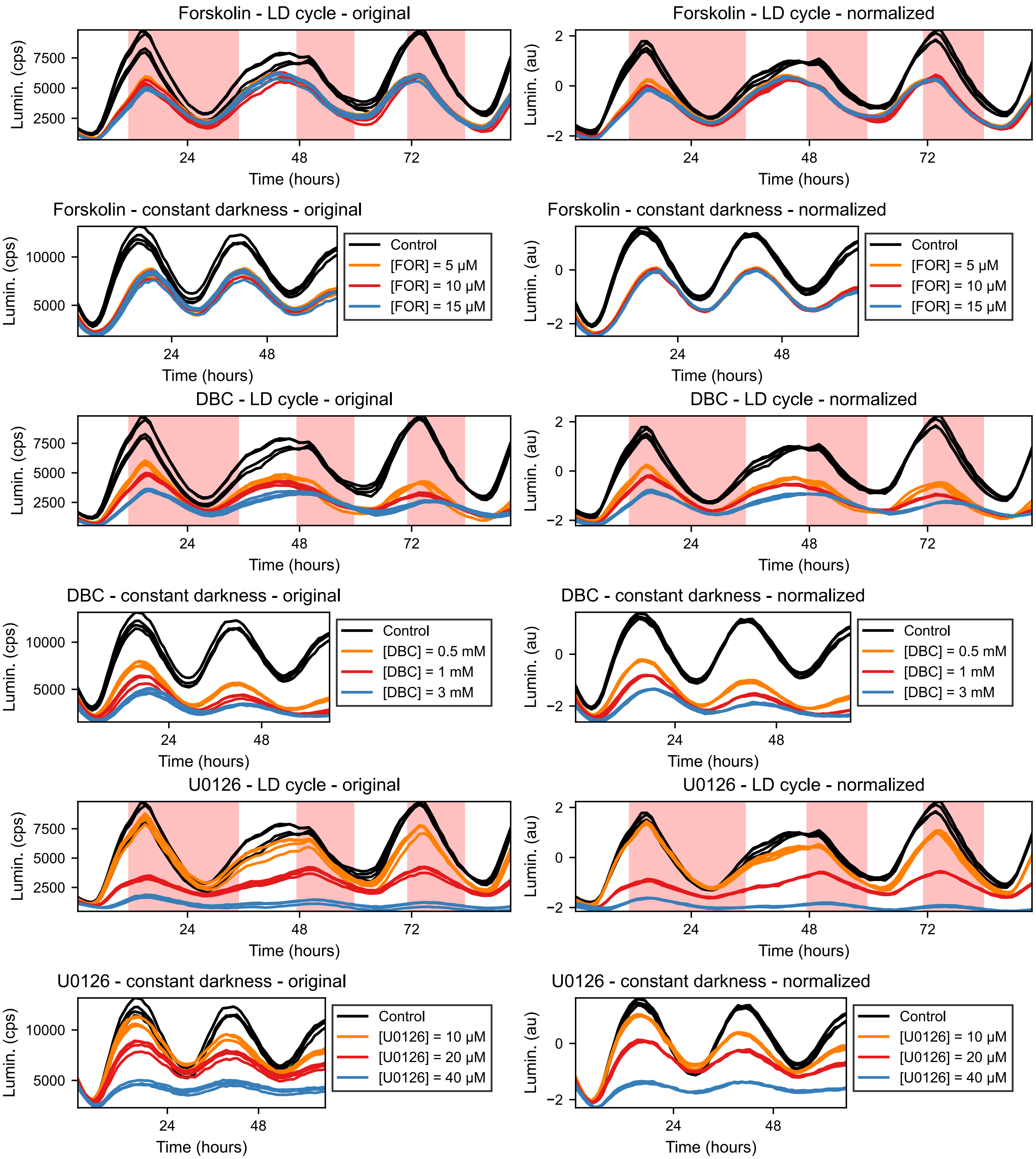

Supplementary Figure S7. Original and normalized data for plate set A. The left column shows original unprocessed traces obtained from pharmacological treatments. In the right column are depicted the same traces after normalization by the adjusted Z-score. The unprocessed traces are shown in counts per second (cps) and the normalized traces are shown in arbitrary units (au).

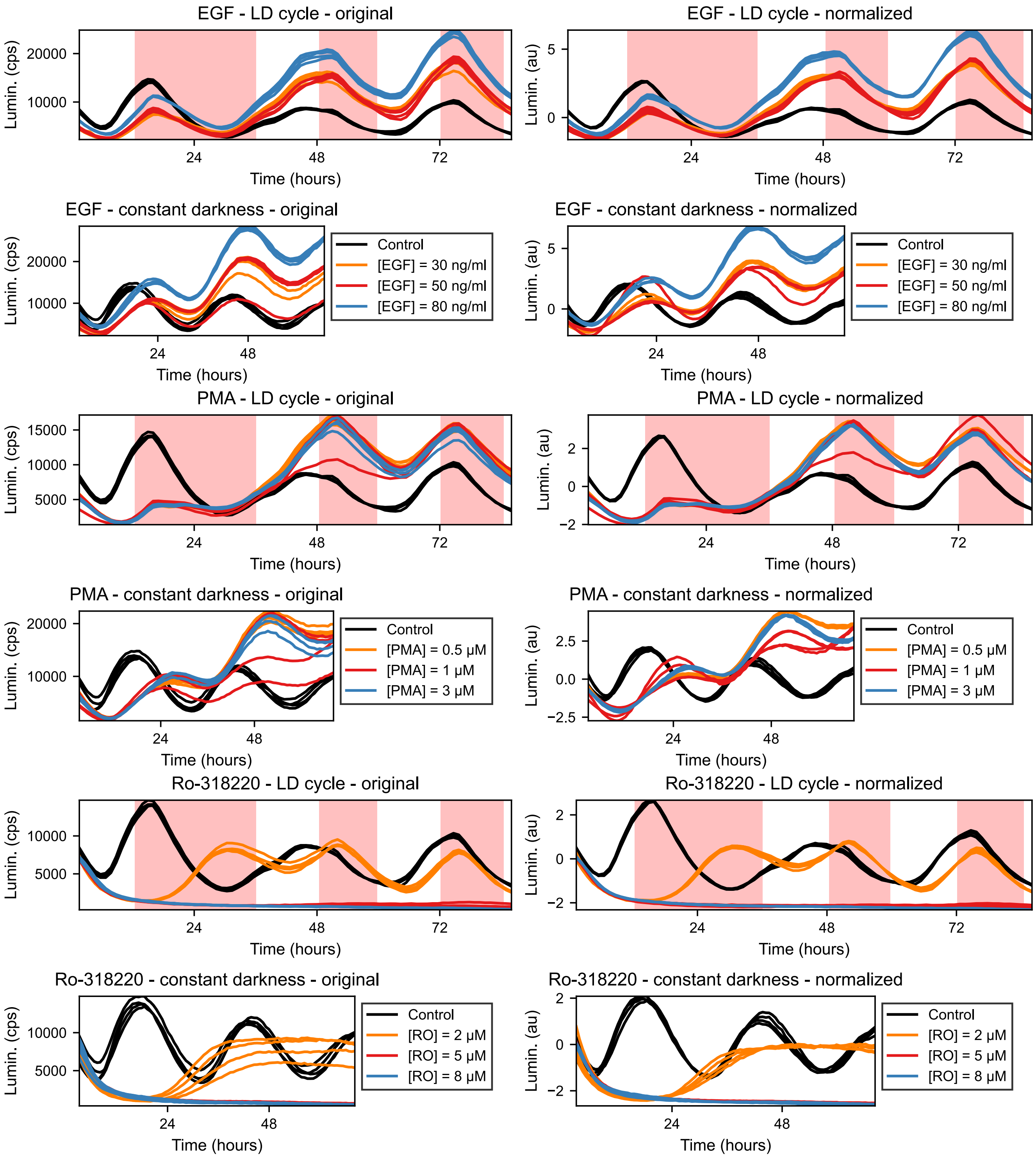

Supplementary Figure S8. Original and normalized traces for plate set B. The left column shows original unprocessed traces obtained from pharmacological treatments. In the right column are depicted the same traces after normalization by the adjusted Z-score. The unprocessed traces are shown in counts per second (cps) and the normalized traces are shown in arbitrary units (au).

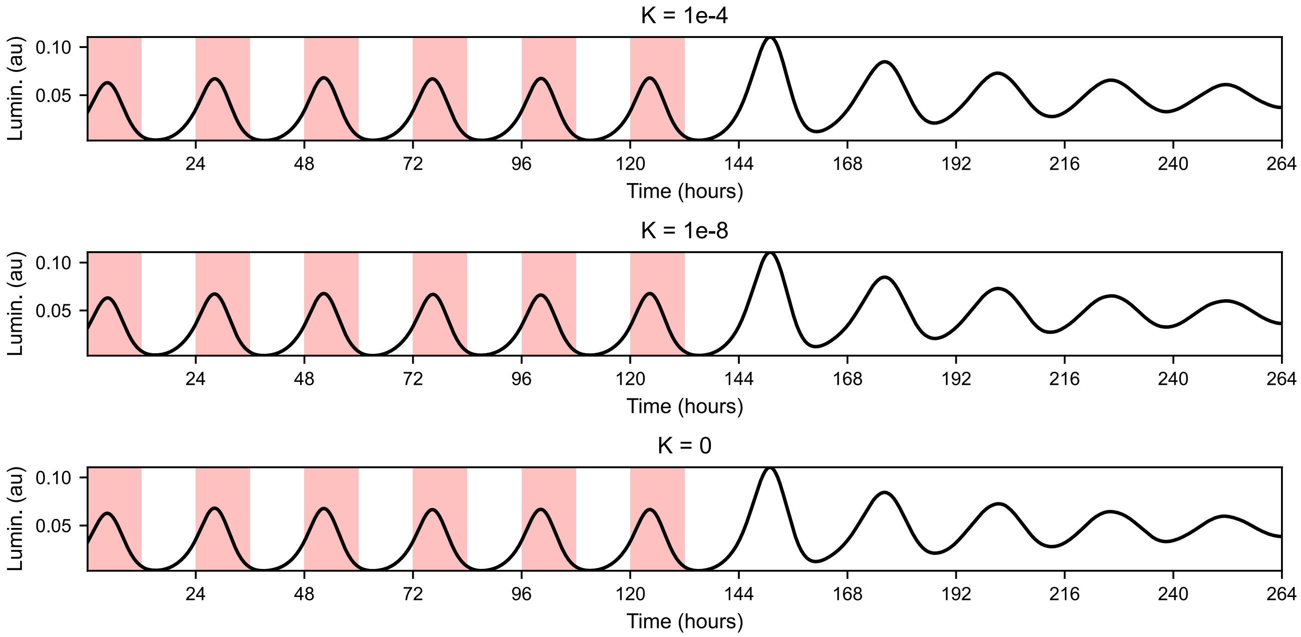

Supplementary Figure S9. Effect of the dissociation constant on model output. We compared the model output for constant values 10^{-4}, 10^{-8}, and 0 (used for our model). Low values are necessary to produce sustained oscillations, however, the model output does not change if a small value for the dissociation constant is approximated by 0.

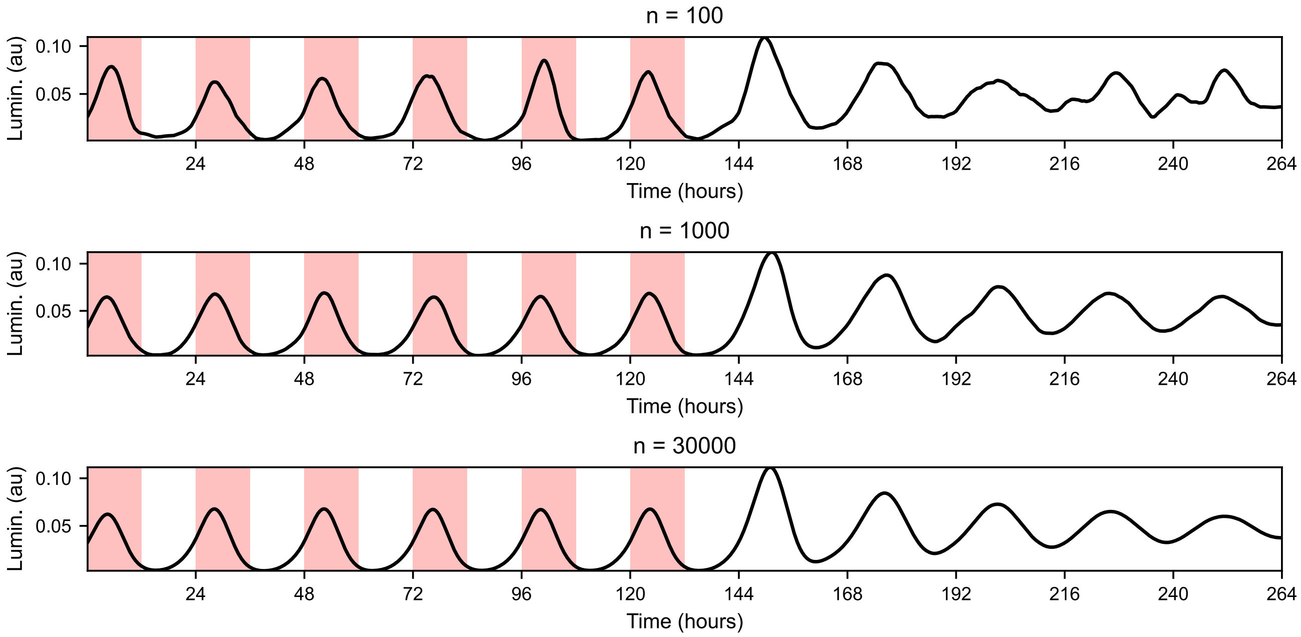

Supplementary Figure S10. Effect of the number of trajectories on the population-level mean. We compared mean values approximated from 100, 1000, and 30000 trajectories. In the biological experiments, a signal from 30000 cells is averaged. Model output presented in the figures is also averaged over 30000 trajectories to be consistent with the experiments. To reduce computational effort, during optimization only 1000 trajectories are used. The difference between 1000 and 30000 trajectories is minimal, indicating that the optimization pipeline produces reliable results.

Supplementary Table S1. Description of well repeats on each plate used in our study. The pharmacological treatments were split into two plate sets (A, B).

Plate	Well repeats
Fitting data	16x untreated
Validation data	4x untreated
15:15 LD cycle	32x untreated
10:10 LD cycle	16x untreated
LD cycle (set A)	4x DMSO control<br>12x FOR (4x 5 μM, 4x 10 μM, 4x 15 μM)<br>12x DBC (4x 0.5 mM, 4x 1 mM, 4x 3 mM)<br>12x U0126 (4x 10 μM, 4x 20 μM, 4x 40 μM)
Constant darkness (set A)	4x DMSO control<br>12x FOR (4x 5 μM, 4x 10 μM, 4x 15 μM)<br>12x DBC (4x 0.5 mM, 4x 1 mM, 4x 3 mM)<br>12x U0126 (4x 10 μM, 4x 20 μM, 4x 40 μM)
LD cycle (set B)	4x DMSO control<br>12x EGF (4x 30 ng/ml, 4x 50 ng/ml, 4x 80 ng/ml)<br>12x PMA (4x 0.5 μM, 4x 1 μM, 4x 3 μM)<br>12x RO (4x 2 μM, 4x 5 μM, 4x 8 μM)
Constant darkness (set B)	4x DMSO control<br>12x EGF (4x 30 ng/ml, 4x 50 ng/ml, 4x 80 ng/ml)<br>12x PMA (4x 0.5 μM, 4x 1 μM, 4x 3 μM)<br>12x RO (4x 2 μM, 4x 5 μM, 4x 8 μM)

Supplementary Table S2. Goodness of fit for pharmacological treatments. The overall fit for a compound is considered good if E_f for both LD cycle and constant darkness are positive (indicated by green color).

Compound	E_f LD cycle	E_f constant darkness
Control A	0.87	0.89
Control B	0.72	0.81
[FOR] = 5 μM	0.74	0.86
[FOR] = 10 μM	0.74	0.87
[FOR] = 15 μM	0.70	0.83
[DBC] = 0.5 mM	0.62	0.73
[DBC] = 1 mM	0.11	0.20
[DBC] = 3 mM	-1.56	-0.20
[U0126] = 10 μM	0.86	0.86
[U0126] = 20 μM	0.68	0.50
[U0126] = 40 μM	-18.19	0.60
[EGF] = 30 ng/ml	0.73	0.73
[EGF] = 50 ng/ml	0.50	0.67
[EGF] = 80 ng/ml	0.69	0.77
[PMA] = 0.5 μM	-1.59	-3.98
[PMA] = 1 μM	0.83	0.89
[PMA] = 3 μM	0.89	0.86
[RO] = 2 μM	0.12	-0.04
[RO] = 5 μM	0.46	0.84
[RO] = 8 μM	0.27	0.84
